# Ambulatory antibiotic prescribing for acute sinusitis: a multicenter, retrospective cohort study evaluating appropriateness

**DOI:** 10.1017/ash.2026.10743

**Published:** 2026-06-25

**Authors:** Kellie Arensman Hannan, Dan Ilges, Kimberly T. Le, Kristin Cole, Ryan W. Stevens, Kelsey Jensen

**Affiliations:** 1 Department of Pharmacy, Mayo Clinic, Rochester, MN, USA; 2 Department of Pharmacy, Mayo Clinic, Phoenix, AZ, USA; 3 Department of Pharmacy Practice and Science, R. Ken Coit College of Pharmacy, The University of Arizona, Tucson, AZ, USA; 4 Division of Clinical Trials and Biostatistics, Mayo Clinic, Rochester, MN, USA; 5 Department of Pharmacy, Mayo Clinic Health System, Austin, MN, USA

## Abstract

**Objective:**

Antibiotics are frequently prescribed for acute sinusitis despite national guidelines recommending antibiotics only if specific symptom criteria are met. We aimed to define the proportion of acute sinusitis encounters meeting criteria for antibiotic prescribing, characterize prescribing practices, and identify factors associated with guideline-discordant prescribing.

**Design:**

This retrospective cohort study included 1,000 randomly selected adult ambulatory encounters with a primary diagnosis of acute sinusitis between January 1, 2024 and March 31, 2024. Encounter notes were reviewed for appropriate antibiotic prescribing criteria as per national guidelines. Encounters were evaluated for drug selection and duration concordance based on local guidelines. A multivariable logistic regression analysis was performed to identify predictors of inappropriate antibiotic prescribing.

**Setting:**

Emergency departments, urgent care centers, and primary care clinics.

**Results:**

Antibiotic prescribing criteria were met for 67.6% of included encounters. Antibiotics were prescribed in 93.5% of encounters that met prescribing criteria, and 80.2% of encounters that did not. Both drug selection and duration were guideline-concordant in 49.2% of total encounters. On multivariable analysis, predictors of inappropriate antibiotic prescribing included cough (OR 2.15, 95% CI 1.08–4.29; *P* = 0.03) and symptom duration between 7 and 9 days (compared to <6 days; OR 7.70, 95% CI 3.24–18.31; *P* < 0.001). Electronic encounters were associated with lower odds of prescribing compared to in-person encounters (OR 0.03, 95% CI 0.01–0.09; *P* < 0.001).

**Conclusions:**

Most encounters for acute sinusitis result in an antibiotic prescription, despite prescribing criteria not being met. These findings may aid antimicrobial stewardship programs in benchmarking and optimizing antibiotic prescribing for acute sinusitis.

## Introduction

Acute sinusitis is a common diagnosis that frequently leads to antibiotic prescriptions in the ambulatory setting,^1^ despite estimations that only 0.5–2% of cases are of bacterial etiology.^2^ Inappropriate use of antibiotics can contribute to adverse effects, antibiotic resistance, and increased health care costs.^3,4^ Acute sinusitis represents a unique challenge for ambulatory antimicrobial stewardship programs (ASP) given that the optimal antibiotic prescribing rate is not well understood, and metrics must include assessment of prescribing appropriateness.^5,6^

The Infectious Diseases Society of America and American Academy of Otolaryngology (AAO) guidelines suggest a conservative approach to antibiotic prescribing in acute sinusitis, recommending antibiotic therapy when sinusitis symptoms have persisted for ≥10 days without clinical improvement, for severe symptoms (ie, high fever AND purulent discharge or facial pain) for three or more days, or with symptom worsening within 10 days after initial improvement (ie, “double worsening”).^7,8^ The AAO guideline additionally recommends watchful waiting for three to five days after initial diagnosis, even if symptoms have persisted but not worsened for 10 or more days.^8^

Despite clinical guidelines recommending against routine utilization of antibiotics for acute sinusitis, antibiotic use remains widespread.^9^ In addition to unnecessary prescribing, opportunities exist regarding agent selection and duration of therapy.^1^ Many factors have been identified in the literature that may contribute to inappropriate prescribing, including but not limited to diagnostic uncertainty, patient expectations, and the pressure on healthcare providers to satisfy patient demands.^10^

The purpose of this study was to evaluate the frequency in which patients with acute sinusitis met criteria for antibiotic prescribing, the appropriateness of prescribing, and, when antibiotics were prescribed, the frequency with which prescribing was guideline-concordant. We also explored patient, encounter, and provider characteristics associated with inappropriate antibiotic prescribing in acute sinusitis, and the frequency of repeat antibiotic prescribing and unplanned respiratory-related repeat healthcare contact within 30 days.

## Methods

This retrospective, observational study included 1,000 randomly selected adult (≥18 yr old) patient encounters for acute sinusitis between January 1, 2024 and March 31, 2024. Random selection was balanced equally among seven geographic regions within our health system (Jacksonville, Florida; Phoenix, Arizona; Southwest Wisconsin; Northwest Wisconsin; Rochester, Minnesota; Southeast Minnesota outside of Rochester; and Southwest Minnesota). Eligible encounters were coded for a primary diagnosis of acute sinusitis (ICD-10 J01.0 [acute maxillary sinusitis], J01.1 [acute frontal sinusitis], J01.2 [acute ethmoidal sinusitis], J01.3 [acute sphenoidal sinusitis], J01.4 [acute pansinusitis], J01.8 [other acute sinusitis], and/or J01.9 [acute sinusitis, unspecified]). Additionally, only encounters performed in-person, electronically (ie, via patient portal without direct patient contact), or via telemedicine (ie, video encounter) at an emergency medicine, urgent care, or primary care Mayo Clinic location were included.

Encounters were excluded if there was a concurrent diagnosis of chronic sinusitis (ICD-10 J32.0 [chronic maxillary sinusitis], J32.1 [chronic frontal sinusitis], J32.2 [chronic ethmoidal sinusitis], J32.3 [chronic sphenoidal sinusitis], J32.4 [chronic pansinusitis], J32.8 [other chronic sinusitis], and/or J32.9 [chronic sinusitis, unspecified]), documented receipt of an ambulatory prescription for a systemic antibiotic in the previous 30 days, other concurrent infection outside of the upper respiratory tract warranting antibiotic therapy as determined via chart review, if the encounter was performed via execution of an institutional acute sinusitis nurse driven protocol, or if the patient was a Minnesota resident who declined to have their records used for research. Repeat encounters for the same patient within the study period were also excluded.

Manual chart abstraction was performed to verify diagnosis, collect baseline patient characteristics, evaluate whether the encounter demonstrated evidence that antibiotic prescribing criteria were met, and collect information on prescribed antibiotics, if applicable. Prescribing an antibiotic was considered to be appropriate if symptoms were documented to have been present for ≥10 days, there was failure to improve ≥7 days after initial diagnosis, severe symptoms were documented (defined as fever ≥102 °F (39.9°C) and either purulent nasal discharge or facial pain) for ≥3 days, or if the clinician documented evidence of double worsening (defined as initial improvement followed by worsening of symptoms). Antibiotic regimens were considered concordant with institutional guidelines in patients prescribed amoxicillin/clavulanate without documented beta-lactam allergy or in patients with documented beta-lactam allergy if prescribed amoxicillin/clavulanate, cefdinir, or doxycycline. Therapy durations of 5–7 days were considered guideline concordant. To assess patient outcomes, we also evaluated unplanned repeat healthcare contact for any respiratory indication and assessed subsequent antibiotic prescribing within 30 days of the index encounter.

Descriptive statistics including frequencies and percentages and medians and interquartile ranges were used to summarize patient, prescriber, and encounter characteristics as well as antibiotic selection and duration. Comparisons were made between those who met/did not meet antibiotic criteria and between those who did/did not have an antibiotic prescribed using χ^2^ tests for categorical data and Wilcoxon rank sum tests for continuous data. For unplanned respiratory-related repeat healthcare contact within 30 days, we calculated 95% exact binomial confidence intervals. Among encounters where criteria for antibiotic prescribing were not met but an antibiotic for immediate initiation (ie, not a watch-and-wait prescription) was prescribed, logistic regression was used to identify patient, encounter, and provider characteristics that were associated with inappropriate antibiotic prescribing. Least absolute shrinkage and selection operator regression was used to select the variables to be included in the multivariable model. Similar methods were used to assess for associations with unplanned repeat healthcare contact within 30 days.

## Results

Of the 1,000 patient encounters included, 676 (67.6%) met antibiotic prescribing criteria (Table 1). Patients were predominantly female (69.5%) with an average age of 50.2 years. Most patients were seen in primary care clinics (70.3%), by an advanced practice provider (65.1%), and at an in-person encounter (70.3%). The most frequently documented symptoms were sinus congestion (80.3%), sinus pressure (62.6%), and cough (55.5%), with a median duration of symptoms documented at the time of the encounter of 14 days.

**Table 1.** Patient, encounter, and provider characteristics Table 1 long description.

	Full cohort (N = 1000)	Met antibiotic criteria (N = 676)	Did not meet antibiotic criteria (N = 324)	*P*-value	Antibiotic prescribed (N = 892)	No antibiotic prescribed (N = 108)	*P*-value
**Department specialty, n (%):**				.81			<.001
Primary care	704 (70.4%)	472 (67.0%)	232 (33.0%)		659 (93.6%)	45 (6.4%)	
Urgent care	254 (25.4%)	174 (68.5%)	80 (31.5%)		195 (76.8%)	59 (23.2%)	
Emergency medicine	42 (4.2%)	30 (71.4%)	12 (28.6%)		38 (90.5%)	4 (9.5%)	
**Primary provider type, n (%):**				.26			.003
Advanced practice provider	651 (65.1%)	448 (68.8%)	203 (31.2%)		567 (87.1%)	84 (12.9%)	
Physician/resident/fellow	349 (34.9%)	228 (65.3%)	121 (34.7%)		325 (93.1%)	24 (6.9%)	
**Encounter type, n (%):**				.004			<.001
In-person	703 (70.3%)	496 (70.6%)	207 (29.4%)		655 (93.2%)	48 (6.8%)	
Telemedicine	216 (21.6%)	126 (58.3%)	90 (41.7%)		197 (91.2%)	19 (8.8%)	
Electronic	81 (8.1%)	54 (66.7%)	27 (33.3%)		40 (49.4%)	41 (50.6%)	
**Age, mean (SD)**	50.2 (16.9)	49.9 (16.9)	50.7 (16.8)	.47	50.8 (17.0)	45.1 (15.4)	.001
**Female sex, n (%)**	695 (69.5%)	466 (67.1%)	229 (32.9%)	.58	610 (87.8%)	85 (12.2%)	.028
**Beta lactam allergy, n (%)**	179 (17.9%)	114 (63.7%)	65 (36.3%)	.22	157 (87.7%)	22 (12.3%)	.48
If yes, severe allergy, n (%) ^*^	76 (42.5%)	47 (61.8%)	29 (38.2%)	.66	65 (85.5%)	11 (14.5%)	.44
**Other concomitant URI, n (%):**				.95			.21
None	968 (96.8%)	654 (67.6%)	314 (32.4%)		863 (89.2%)	105 (10.8%)	
Otitis Media	21 (2.1%)	14 (66.7%)	7 (33.3%)		20 (95.2%)	1 (4.8%)	
Pharyngitis	5 (0.5%)	4 (80.0%)	1 (20.0%)		5 (100.0%)	0 (0.0%)	
Other	6 (0.6%)	4 (66.7%)	2 (33.3%)		4 (66.7%)	2 (33.3%)	
**Symptoms documented, n (%):**							
Sinus congestion	801 (80.3%)	546 (68.2%)	255 (31.8%)	.44	711 (88.8%)	90 (11.2%)	.41
Sinus pressure	624 (62.6%)	423 (67.8%)	201 (32.2%)	.87	554 (88.8%)	70 (11.2%)	.61
Rhinorrhea	433 (43.4%)	300 (69.3%)	133 (30.7%)	.32	381 (88.0%)	52 (12.0%)	.29
Fever	95 (9.5%)	58 (61.1%)	37 (38.9%)	.15	88 (92.6%)	5 (7.4%)	.25
Fatigue	101 (10.1%)	76 (75.2%)	25 (24.8%)	.083	96 (95.0%)	5 (5.0%)	.045
Headache	352 (35.3%)	248 (70.5%)	104 (29.5%)	.16	300 (85.2%)	52 (14.8%)	.003
Face pain/discomfort	414 (41.5%)	298 (72.0%)	116 (28.0%)	.013	365 (88.2%)	49 (11.8%)	.39
Cough	553 (55.5%)	379 (68.5%)	174 (31.5%)	.48	507 (91.7%)	46 (8.3%)	.004
Other	341 (34.2%)	222 (65.1%)	119 (34.9%)	.22	302 (88.6%)	39 (11.4%)	.66
None of the above	1 (0.1%)	0 (0.0%)	1 (100.0%)	.15	1 (100.0%)	0 (0.0%)	.73
**Duration of symptoms (days), median (IQR):**	14 (7, 21)	14 (12, 21)	7 (4, 7)	<.001	14 (8, 21)	7 (4, 14)	<.001
≤6 days, n (%)	151 (15.8%)	16 (10.6%)	135 (89.4%)		103 (68.2%)	48 (31.8%)	
7-9 days, n (%)	183 (19.2%)	33 (18.0%)	150 (82.0%)		158 (86.3%)	25 (13.7%)	
10-14 days, n (%)	324 (34.0%)	324 (100.0%)	0 (0.0%)		307 (94.8%)	17 (5.2%)	
15-28 days, n (%)	179 (18.8%)	179 (100.0%)	0 (0.0%)		168 (93.9%)	11 (6.1%)	
>28 days, n (%)	116 (12.2%)	116 (100.0%)	0 (0.0%)		113 (97.4%)	3 (2.6%)	
**Antibiotic prescribing criteria met**, **n (%):**							
Duration >/= 10 days	620 (65.1%)	620 (100.0%)	–	<.001	588 (94.8%)	32 (5.2%)	<.001
Failure to improve after 7 days	21 (2.1%)	21 (100.0%)	–	<.001	18 (85.7%)	3 (14.3%)	.60
Severe symptoms	85 (8.5%)	85 (100.0%)	–	<.001	73 (85.9%)	12 (14.1%)	.30
Double worsening	115 (11.5%)	115 (100.0%)	–	<.001	107 (93.0%)	8 (7.0%)	.16
**Unplanned repeat healthcare contact, n (%)**	128 (12.8%)	88 (68.8%)	40 (31.3%)	.77	106 (82.8%)	22 (17.2%)	.013
Time (days) to repeat contact, median (IQR)	12 (6, 21)	12 (6, 21)	12 (9, 20)	.36	12 (7, 21)	8 (1, 15)	.022
Type of repeat healthcare contact, n (%)				.61			.016
Clinic encounter	92 (71.9%)	62 (67.4%)	30 (32.6%)		81 (88.0%)	11 (12.0%)	
ED encounter	15 (11.7%)	12 (80.0%)	3 (20.0%)		12 (80.0%)	3 (20.0%)	
Telemedicine	21 (16.4%)	14 (66.7%)	7 (33.3%)		13 (61.9%)	8 (38.1%)	

* Severe allergies were defined as allergies with documented chart reactions consistent with a severe cutaneous adverse reaction (ie, drug rash with eosinophilia and systemic symptoms, Stevens-Johnson syndrome, toxic epidermal necrolysis syndrome) or an IgE-mediated allergic reaction (ie, anaphylaxis, urticaria, or angioedema).

An antibiotic prescription was issued in 892 (89.2%) of included encounters. Of the 892 patients who received antibiotics, prescribing criteria were met in 632 (70.9%) encounters and were not met in 260 (29.1%) encounters. Conversely, of the 108 patients who did not receive antibiotics, 44 (40.7%) met prescribing criteria but were not treated. Of those 44 patients who were not treated, 8 (18.2%) had unplanned respiratory-related repeat healthcare contact within 30 days.

Among the 704 patients seen in primary care clinics, 93.6% received antibiotics, though only 67% met prescribing criteria. Emergency medicine showed a similar trend, with 90.5% of 42 encounters receiving antibiotics despite just 71.4% meeting criteria. Notably, prescribing rates were lower in urgent care encounters, where 76.8% of 254 patients received antibiotics, though only 68.5% of patients met criteria. Furthermore, antibiotics were prescribed less frequently by advanced practice providers (87.1% of encounters) compared to physicians, residents, or fellows (93.1%), despite prescribing criteria being met in only 68.8% and 65.3% of encounters, respectively.

Antibiotic prescribing patterns varied across encounter types. Of the 703 patients seen during in-person encounters, 93.2% received antibiotics, although only 70.6% met prescribing criteria. Telemedicine encounters showed an even stronger tendency toward prescribing, with 91.2% of 216 patients receiving antibiotics despite just 58.3% meeting criteria. In contrast, electronic encounters had the lowest prescribing rates with only 49.4% of 81 receiving antibiotics despite 66.7% meeting prescribing criteria.

The most frequently prescribed antibiotics were amoxicillin/clavulanate (60.9%) or doxycycline (19.1%), with median duration of therapy of 7 days (Table 2). A duration of therapy longer than 7 days was prescribed in 35.4% (316/892) of encounters. Overall guideline concordance, with respect to both drug selection and duration, was observed in 49.2% (439/892) of the overall cohort without evidence of a statistically significant difference between those meeting prescribing criteria and those who did not. Although there was no statistically significant difference in guideline-concordant duration of therapy observed between patients who met antibiotic prescribing criteria and those who did not, guideline-concordant drug selection was significantly less frequent among patients who did not meet the criteria (66.5% vs 77.2%, *P* = .001). This appears to have been predominantly driven by a decrease in the prescribing of amoxicillin/clavulanate and an increase in azithromycin prescribing. A watch-and-wait prescribing strategy was infrequently used (6.8%) in the overall cohort.

**Table 2.** Antibiotic prescription characteristics Table 2 long description.

	Total encounters with an antibiotic prescribed (N = 892)	Antibiotic prescribing criteria met (N = 632)	Antibiotic prescribing criteria not met (N = 260)	*P*-value
**Watch-and-wait prescription, n (%)**	61 (6.8%)	33 (5.2)	28 (10.8)	.003
Watch-and-wait prescription filled, n (%):	–	–	–	.45
Yes	15 (24.6%)	9 (27.3%)	6 (21.4%)	–
No	4 (6.6%)	1 (3.0%)	3 (10.7%)	–
Unknown	42 (68.9%)	23 (69.7%)	19 (67.9%)	–
**Antibiotic prescribed, n (%):**				
Amoxicillin/clavulanate	543 (60.9%)	412 (65.2%)	131 (50.4%)	<.001
Doxycycline	170 (19.1%)	111 (17.6%)	59 (22.7%)	.076
Amoxicillin	69 (7.7%)	43 (6.8%)	26 (10.0%)	.10
Azithromycin	55 (6.2%)	29 (4.6%)	26 (10.0%)	.002
Cefdinir	40 (4.5%)	29 (4.6%)	11 (4.2%)	.81
Other	11 (1.2%)	7 (1.1%)	4 (1.5%)	.59
Levofloxacin	4 (0.4%)	1 (0.2%)	3 (1.2%)	.043
Trimethoprim/sulfamethoxazole	2 (0.2%)	2 (0.3%)	0 (0.0%)	.36
**Guideline concordant prescription, n (%)**	439 (49.2%)	323 (51.1%)	116 (44.6%)	.078
95% CI	45.9%–52.5%	47.1%–55.1%	38.5%–50.9%	–
Guideline concordant drug selection, n (%)	661 (74.1%)	488 (77.2%)	173 (66.5%)	.001
Guideline concordant duration, n (%)	575 (64.5%)	405 (64.1%)	170 (65.4%)	.71
**Duration of therapy (days), median (IQR)**	7 (5, 10)	7 (5, 10)	7 (5, 10)	.64

When antibiotic prescribing criteria were met, cases usually involved symptom duration ≥10 days (65.1%), followed by double worsening (11.5%), and severe symptoms (8.5%) (Table 1). Logistic regression was used to identify predictors of antibiotic prescribing among encounters where antibiotic prescribing criteria were not met (ie, inappropriate prescribing) (Table 3). Univariate analysis identified advancing age (per 10 yr), physician/resident/fellow provider type, symptoms of headache or cough, and symptom duration not documented or 7–9 days as predictors of inappropriate prescribing. Electronic encounters and encounters seen in urgent care centers were associated with lower odds of inappropriate prescribing compared with in-person and primary care encounters, respectively. Multivariable logistic regression identified cough (OR 2.15, 95% CI 1.08–4.29; *P* = .03), duration of symptoms between 7 and 9 days (compared to < 6 d; OR 7.70, 95% CI 3.24–18.31; *P* < .001), and duration of symptoms not documented (compared to <6 days; OR 5.01, 95% CI (1.41–17.82; *P* = .013) as predictors of inappropriate antibiotic prescribing. Electronic encounters were associated with lower odds of inappropriate antibiotic prescribing compared to in-person encounters (OR 0.03, 95% CI 0.01–0.09; *P* < .001).

**Table 3.** Predictors of antibiotic prescribing among encounters where antibiotic prescribing criteria were not met Table 3 long description.

	Univariate		Multivariable	
	Odds ratio (95% CI)	*P*-value	Odds ratio (95% CI)	*P*-value
**Encounter type:**				
In-person	Reference	–	Reference	–
Telemedicine	1.07 (0.53–2.16)	.86	1.57 (0.73–3.40)	.25
Electronic	0.04 (0.01–0.11)	<.001	0.03 (0.01–0.09)	<.001
**Age (per 10 years)**	1.29 (1.09–1.53)	.003	–	–
**Sex:**				
Female	Reference	–	–	–
Male	1.41 (0.74–2.68)	.29	–	–
**Department specialty:**				
Primary Care	Reference	–	–	–
Urgent Care	0.16 (0.09–0.29)	<.00	–	–
Emergency	0.69 (0.14–3.36)	.65	–	–
**Primary provider type:**				
Advanced practice provider	Reference	–	–	–
Physician/resident/fellow	2.57 (1.34–4.90)	.004	–	–
**Beta lactam allergy**	0.91 (0.46–1.78)	.78	–	–
**Symptoms documented:**				
Sinus congestion	0.65 (0.31–1.37)	.26	–	–
Sinus pressure	0.82 (0.46–1.47)	.50	–	–
Rhinorrhea	0.59 (0.34–1.03)	.062	–	–
Fever	1.39 (0.55–3.50)	.49	–	–
Fatigue	3.10 (0.71–13.58)	.13	–	–
Headache	0.45 (0.26–0.80)	.007	–	–
Face pain/discomfort	0.95 (0.54–1.69)	.87	–	–
Cough	2.55 (1.44–4.53)	.001	2.15 (1.08–4.29)	.030
Other	1.02 (0.57–1.81)	.95	–	–
**Duration of symptoms**				
≤ 6 days	Reference	–	Reference	–
7–9 days	4.48 (2.37–8.48)	<.00	7.70 (3.24–18.31)	<.00
Not documented	6.91 (2.01–23.76)	.002	5.01 (1.41–17.82)	.013

Unplanned respiratory-related repeat healthcare contact within 30 days was more common after encounters where an antibiotic was not prescribed (20.4% vs 11.9%, *P* = .013) (Table 1). Patients who did not receive antibiotics sought repeat healthcare contact sooner than those who did, at a median of 8 days versus 12 days, respectively. Logistic regression was used to identify predictors of unplanned repeat healthcare contact (Table 4). On univariate analysis, index care provided via an electronic encounter (compared to in person encounters; OR 2.82, 95% CI 1.64–4.85; *P* < .001) and care provided in the emergency department (compared to primary care; OR 1.68, 95% CI 1.12–2.54; *P* = .011) were identified as risk factors for 30-day unplanned repeat contact, whereas a duration of symptoms >28 days and antibiotic prescription provided at the index encounter were associated with less unplanned repeat contact (compared to <6 d; OR 0.40, 95% CI 0.18–0.90; *P* = .027). The only characteristic that remained a statistically significant predictor in the multivariable model was index care provided via electronic encounter type (OR 2.25, 95% CI 1.15–4.42; *P* = .019).

**Table 4.** Predictors of unplanned repeat healthcare contact within 30 days Table 4 long description.

	Univariate		Multivariable	
	Odds ratio (95% CI)	*P*-value	Odds ratio (95% CI)	*P*-value
**Encounter type:**				
In-person	Reference	–	Reference	–
Telemedicine	0.95 (0.58–1.53)	.82	0.98 (0.60–1.60)	.92
Electronic	2.82 (1.64–4.85)	<.001	2.25 (1.15–4.42)	.019
**Age (per 10 years)**	0.95 (0.85–1.06)	.40	–	–
**Male sex**	0.80 (0.53–1.22)	.30	–	–
**Department specialty:**				
Primary Care	Reference		Reference	–
Urgent Care	1.34 (0.55–3.27)	.53	1.20 (0.74–1.95)	.46
Emergency	1.68 (1.12–2.51)	.011	1.33 (0.54–3.29)	.54
**Primary provider type:**				
Advanced practice provider	Reference		–	–
Physician/Resident/Fellow	1.10 (0.75–1.61)	.64	–	–
**Beta lactam allergy**	1.34 (0.85–2.11)	.21	–	–
**Symptoms documented:**				
Sinus congestion	0.89 (0.57–1.41)	.63	–	–
Sinus pressure	0.88 (0.60–1.28)	.49	–	–
Rhinorrhea	0.86 (0.59–1.25)	.43	–	–
Fever	1.44 (0.81–2.56)	.21	–	–
Fatigue	0.65 (0.32–1.31)	.23	–	–
Headache	1.22 (0.83–1.79)	.31	–	–
Face pain/discomfort	1.09 (0.75–1.58)	.66	–	–
Cough	0.92 (0.63–1.33)	.64	–	–
Other	1.06 (0.72–1.57)	.75	–	–
**Duration of symptoms (days):**				
≤6 days	Reference	–	–	–
7–9 days	0.59 (0.32–1.11)	.10	–	–
10–14 days	0.70 (0.41–1.19)	.18	–	–
15–28 days	0.85 (0.47–1.54)	.60	–	–
>28 days	0.40 (0.18–0.90)	.027	–	–
Not documented	0.57 (0.21–1.59)	.28	–	–
**Antibiotic prescribing criteria met**	1.06 (0.71–1.59)	.77	–	–
**Antibiotic prescribed**	0.53 (0.32–0.88)	.014	0.77 (0.439–1.37)	.37

## Discussion

This was a large retrospective evaluation of antibiotic prescribing appropriateness for acute sinusitis. We found that 89.2% of ambulatory encounters for acute sinusitis resulted in an antibiotic prescription, despite antibiotic prescribing criteria only being met in 67.6% of encounters. An antibiotic was prescribed at 93.5% of encounters that met prescribing criteria versus 80.2% of encounters that did not.

Previous studies have demonstrated that guideline-based criteria are infrequently leveraged to inform antibiotic decisions. Although national data indicate that antibiotics are prescribed in 72% of acute sinusitis encounters,^11^ a study by Vazquez Deida *et al*., analyzing insurance claims data reported a higher prescribing rate of 82%.^1^ In a retrospective review by Truitt *et al.,* among 425 acute sinusitis encounters, 50% of encounters did not meet prescribing criteria.^9^ Nevertheless, antibiotics were prescribed in 95% of the overall cohort, a figure higher than the rate identified in our study.

When clinical criteria for antibiotic prescribing in sinusitis are met for adult patients, and in the absence of complicating factors, guidelines recommend use of amoxicillin, amoxicillin/clavulanate or doxycycline as first-line or the use of levofloxacin or moxifloxacin as second-line alternatives for a total duration of 5–7 days.^7,8^ In the study conducted by Vazquez Deida *et al.*, 51.9% of patients received first-line agents for a median of 10 days.^1^ Despite some differences in inclusion criteria (eg, our inclusion of patients who were immunocompromised or over the age of 60), 74.1% of patients in our study received guideline-concordant drug selection. Azithromycin (6.2% vs 20%) and cefdinir (4.5% vs 6%) were used less frequently in our cohort, and median duration of therapy was shorter at 7 days compared with 10 days. Despite these differences, the rate of overall guideline-concordance (ie, drug selection and duration) for our cohort was low at 49%.

Antibiotic prescribing rates were lower during electronic encounters compared to other encounter types. A retrospective cohort study by Johnson *et al.* demonstrated that patients who completed electronic encounters for acute sinusitis were less likely to receive antibiotics compared to in-person encounters (68.6% vs 94.3%; *P* < .001).^12^ Among our cohort, electronic encounters had the lowest rate of antibiotic prescribing, with only 49.4% of patients receiving antibiotics. Though we cannot draw casual inferences, this trend suggests that electronic encounters may be associated with a more judicious antibiotic prescribing in acute sinusitis based on training, guideline awareness, and comfort with diagnostic uncertainty.^13,14^ Patient attitudes and expectations may also vary across settings and encounter types. During electronic encounters, patients may have lower expectations for antibiotics and may prioritize convenience and symptom relief over receipt of antibiotics.^15^ Providers may experience less pressure to prescribe antibiotics and/or more easily decline antibiotic prescribing when communicating with patients via indirect methods.^13^ Lastly, some clinical workflows may inherently promote non-prescribing secondary to clinical decision support, limited physical examination capabilities increasing reliance on structured questionnaires, and more stringent documentation requirements on virtual platforms.^12,14–16^

Among encounters where prescribing criteria were met for acute sinusitis, symptom duration of ≥10 days was used as justification for 65.1%. This finding highlights a reliance on prolonged duration of symptoms as justification for antibiotic use, compared with other features like double worsening and severe symptoms that occur less frequently. Early publications on acute sinusitis suggested that symptoms >7 days may suggest bacterial superinfection.^17^ However, more contemporary data suggest that symptoms from a viral illness can last much longer. For example, the median duration of symptoms for adults with COVID-19 was 15 (IQR 8–24) days.^18^ The predominance of symptom duration in clinical decision-making underscores the need for enhanced provider education and decision support, particularly in recognizing that viral infections may persist beyond commonly anticipated timeframes. The expansion of rapid molecular diagnostics for upper respiratory tract infection (URI) in the ambulatory space may help further our understanding of anticipated duration of symptoms by viral pathogen.^19^ Given that the updated 2025 AAO guideline extended the recommendation for watchful waiting as initial management for all patients with uncomplicated acute bacterial sinusitis including in patients with symptoms persisting beyond 10 days without worsening, symptom duration appears to be an area ripe for antimicrobial stewardship and education.^8^

Previous studies have noted that the presence of certain symptoms, including prolonged illness and purulent discharge, have been associated with antibiotic prescribing.^20,21^ In our study, multivariable logistic regression identified presence of cough and undocumented symptom duration or symptom duration of 7–9 days as independent predictors of inappropriate prescribing. These findings highlight challenges in distinguishing between bacterial and viral etiologies based on symptomatic presentation, which are likely compounded by inconsistencies in documentation.^7^ An additional diagnostic challenge is that patients with sinusitis symptoms may have noninfectious etiologies such as rhinitis.^22,23^

Although respiratory-related repeat healthcare contact was more common after encounters when an antibiotic was not prescribed, antibiotic prescribing was not found to have a significant protective effect against unplanned respiratory-related repeat healthcare contact in multivariable logistic regression modeling. Electronic encounters were associated with more unplanned respiratory-related repeat healthcare contact than in-person encounters, aligning with trends reported in existing literature.^12^ We hypothesize that this may have been influenced by less frequent antibiotic prescribing, as well as the inherent limitations presented by the lack of direct patient-provider interactions during this type of encounter.

Ambulatory ASP efforts aiming to improve antibiotic prescribing for respiratory tract infections have primarily focused on syndromes where antibiotics are never appropriate (ie, Tier 3 syndromes) rather than syndromes where antibiotics are sometimes appropriate (Tier 2 syndromes), such as acute sinusitis.^11,24^ Given the high volume of encounters and commonality of durations of therapy exceeding 7 days, future studies should focus on optimizing antibiotic prescribing (ie, selection and duration) and evaluating interventions that enhance identification of patients who may benefit from antibiotics in Tier 2 syndromes. Although Tier 2 syndromes are more challenging to study than Tier 3 syndromes due to nuanced decision-making and data complexity, they represent a compelling and relatively untapped opportunity to advance antimicrobial stewardship given high encounter frequency and substantial potential for improvement.

Our study is limited by its retrospective design and by potential misclassification bias given reliance on provider documentation of patient-reported symptoms within progress notes. The use of ICD-10 codes to identify acute sinusitis encounters for inclusion may have resulted in unintentional omission or unintentional inclusion of sinusitis encounters miscoded as either acute or chronic. Furthermore, use of ICD-10 codes may have biased rates of satisfaction of prescribing criteria if providers favored use of sinusitis-specific codes in cases where antibiotic prescribing criteria were met and alternative respiratory diagnosis codes in more clinically ambiguous cases. Included encounters occurred between January and March of a single year to align with our typical respiratory viral illness season. This seasonality may have led to the capture of heightened prescribing behaviors; however, our team selected this time frame to capture a snapshot of prescribing practices during which providers would be seeing the highest number of encounters for sinusitis and other URIs. Lastly, due to inclusion of multiple department specialties across multiple regions of our healthcare enterprise, we were unable to account for variability in triage and scheduling characteristics at the individual practice level. As such, it is important to acknowledge that unique triage practices employed by specific regions, clinics, or specialties may have influenced the overall rate at which prescribing criteria were met. This variability may limit the external validity of these findings to other health systems or practice settings.

In conclusion, roughly one-third of patients presenting with symptoms of sinusitis failed to meet antibiotic prescribing criteria. Despite symptom-based prescribing criteria not being met, antibiotics were frequently prescribed for treatment of acute sinusitis. ASP targets for acute sinusitis may include frequency of antibiotic prescribing and/or duration of therapy in department specialties and encounter types with poor performance for these metrics with careful attention to counterbalance measures such as repeat healthcare contact.

## Table 1. Long description

The table presents data on one thousand patient encounters, categorizing them based on whether they met antibiotic prescribing criteria. It includes details on department specialty, primary provider type, encounter type, age, sex, beta-lactam allergy, other concomitant URI, symptoms documented, duration of symptoms, antibiotic prescribing criteria met, unplanned repeat healthcare contact, and type of repeat healthcare contact. The table has thirteen columns and twenty rows, with notable trends including a higher percentage of patients meeting antibiotic criteria in urgent care and telemedicine encounters, as well as a predominance of sinus congestion and sinus pressure symptoms.

Navigate back to Table 1..

## Table 2. Long description

The table presents data on antibiotic prescription characteristics, focusing on encounters where antibiotics were prescribed. It includes columns for total encounters, encounters meeting antibiotic prescribing criteria, and those not meeting the criteria. The table has 12 rows and 7 columns, detailing watch-and-wait prescriptions, types of antibiotics prescribed, and guideline concordance. Notable trends include the frequent prescription of amoxicillin/clavulanate and doxycycline, with a median therapy duration of 7 days. Guideline concordance for drug selection and duration is also analyzed, showing variations between encounters that met and did not meet prescribing criteria. The watch-and-wait prescription strategy was used infrequently.

Navigate back to Table 2..

## Table 3. Long description

A table with three columns and multiple rows, comparing predictors of antibiotic prescribing among encounters where criteria were not met. The columns include univariate odds ratio with confidence intervals and P-value, and multivariable odds ratio with confidence intervals and P-value. The table lists various factors such as encounter type, age, sex, department specialty, primary provider type, beta lactam allergy, symptoms documented, and duration of symptoms. Notable trends include lower odds of inappropriate prescribing for electronic encounters and urgent care departments, and higher odds for symptoms like cough and longer symptom duration.

Navigate back to Table 3..

## Table 4. Long description

A table with 22 rows and 8 columns comparing predictors of unplanned repeat healthcare contact within 30 days. The table includes univariate and multivariable analysis with odds ratios and P-values for various factors such as encounter type, age, sex, department specialty, primary provider type, symptoms documented, duration of symptoms, antibiotic prescribing criteria, and antibiotic prescribed. Notable findings include electronic encounters having a higher odds ratio for repeat contact in both univariate and multivariable analyses.

Navigate back to Table 4..

## References

[1] Vazquez Deida AA , Bizune DJ , Kim C , et al. Opportunities to improve antibiotic prescribing for adults with acute sinusitis, United States, 2016–2020. Open Forum Infect Dis 2024;11:ofae420. 39100530 10.1093/ofid/ofae420 PMC11297501

[2] Fokkens WJ , Lund VJ , Mullol J , et al. EPOS 2012: European position paper on rhinosinusitis and nasal polyps 2012. A summary for otorhinolaryngologists. Rhinology 2012;50:1–12. 22469599 10.4193/Rhino12.000

[3] Shehab N , Patel PR , Srinivasan A , Budnitz DS. Emergency department visits for antibiotic-associated adverse events. Clin Infect Dis 2008;47:735–743. 18694344 10.1086/591126

[4] Friedman ND , Temkin E , Carmeli Y. The negative impact of antibiotic resistance. Clin Microbiol Infect 2016;22:416–422. 26706614 10.1016/j.cmi.2015.12.002

[5] Stenehjem E , Wallin A , Fleming-Dutra KE , et al. Antibiotic prescribing variability in a large urgent care network: a new target for outpatient stewardship. Clin Infect Dis 2020;70:1781–1787. 31641768 10.1093/cid/ciz910 PMC7768670

[6] Ilges D , Jensen K , Draper E , et al. Evaluation of multisite programmatic bundle to reduce unnecessary antibiotic prescribing for respiratory infections: a retrospective cohort study. Open Forum Infect Dis 2023;10:ofad585. 38111752 10.1093/ofid/ofad585 PMC10727194

[7] Chow AW , Benninger MS , Brook I , et al. IDSA clinical practice guideline for acute bacterial rhinosinusitis in children and adults. Clin Infect Dis 2012;54:e72–e112. 22438350 10.1093/cid/cir1043

[8] Payne SC , McKenna M , Buckley J , et al. Clinical practice guideline: adult sinusitis update. Otolaryngol--head neck surg 2025;173:S1–S56. 40742114 10.1002/ohn.1344

[9] Truitt KN , Brown T , Lee JY , Linder JA. Appropriateness of antibiotic prescribing for acute sinusitis in primary care: a cross-sectional study. Clin Infect Dis 2021;72:311–314. 33501972 10.1093/cid/ciaa736 PMC7840109

[10] Teixeira Rodrigues A , Roque F , Falcão A , Figueiras A , Herdeiro MT. Understanding physician antibiotic prescribing behaviour: a systematic review of qualitative studies. Int J Antimicrob Agents 2013;41:203–212. 23127482 10.1016/j.ijantimicag.2012.09.003

[11] Fleming-Dutra KE , Hersh AL , Shapiro DJ , et al. Prevalence of inappropriate antibiotic prescriptions among US ambulatory care visits, 2010-2011. JAMA 2016;315:1864–1873. 27139059 10.1001/jama.2016.4151

[12] Johnson KM , Dumkow LE , Burns KW , Yee MA , Egwuatu NE. Comparison of diagnosis and prescribing practices between virtual visits and office visits for adults diagnosed with sinusitis within a primary care network. Open Forum Infect Dis 2019;6:ofz393. 31660415 10.1093/ofid/ofz393 PMC6778270

[13] Penza KS , Murray MA , Myers JF , Furst JW , Pecina JL. Management of acute sinusitis via e-visit. Telemed J E Health 2021;27:532–536. 32522103 10.1089/tmj.2020.0047

[14] Wasylyshyn AI , Kaye KS , Chen J , et al. Improving antibiotic use for sinusitis and upper respiratory tract infections: a virtual-visit antibiotic stewardship initiative. Infect Control Hosp Epidemiol 2022;43:1890–1893. 35094721 10.1017/ice.2022.19 PMC8828391

[15] Kim S , Thombley R , Eiden E , et al. Differences in physician electronic health record use by telemedicine intensity: evidence from 2 academic medical centers. J Am Med Inform Assoc 2025;32:1462–1470. 40646683 10.1093/jamia/ocaf122 PMC12361853

[16] Dorsey ER , Topol EJ. State of telehealth. N Engl J Med 2016;375:154–161. 27410924 10.1056/NEJMra1601705

[17] Gwaltney JM. Acute community-acquired sinusitis. Clin Infect Dis 1996;23:1209–1223. quiz 1224–1225. 8953061 10.1093/clinids/23.6.1209 PMC7109987

[18] Lane A , Hunter K , Lee EL , et al. Clinical characteristics and symptom duration among outpatients with COVID-19. Am J Infect Control 2022;50:383–389. 34780804 10.1016/j.ajic.2021.10.039 PMC8590478

[19] Lapin K. Retrospective case study of respiratory viral panel PCR testing in primary care. J Family Med Prim Care 2024;13:388–392. 38482294 10.4103/jfmpc.jfmpc_446_23 PMC10931873

[20] Rosenfeld RM , Piccirillo JF , Chandrasekhar SS , et al. Clinical practice guideline (Update): adult sinusitis. Otolaryngol Head Neck Surg 2015;152:S1–S39. 10.1177/0194599815572097 25832968

[21] Lemiengre MB , Driel M L van , Merenstein D , Liira H , Mäkelä M , Sutter AID. Antibiotics for acute rhinosinusitis in adults. Cochrane Database Syst Rev 2018;9:CD006089. 30198548 10.1002/14651858.CD006089.pub5 PMC6513448

[22] Hornung CM , Ganti A , Lunos S , Tyler MA. Characterizing trends in diagnosis and management of sinusitis in a large health care system: from primary care to otolaryngology. Ann Otol Rhinol Laryngol 2024;133:476–484. 38345045 10.1177/00034894241230365

[23] Pynnonen MA , Terrell JE. Conditions that masquerade as chronic rhinosinusitis: a medical record review. Arch Otolaryngol Head Neck Surg 2006;132:748–751. 16847183 10.1001/archotol.132.7.748

[24] Stenehjem E , Wallin A , Willis P , et al. Implementation of an antibiotic stewardship initiative in a large urgent care network. JAMA Netw Open 2023;6:e2313011. 37166794 10.1001/jamanetworkopen.2023.13011 PMC10176123

